# Analysis of Demographic and Socioeconomic Factors Influencing Adherence to a Web-Based Intervention Among Patients After Acute Coronary Syndrome: Prospective Observational Cohort Study

**DOI:** 10.2196/57058

**Published:** 2024-08-02

**Authors:** Biagio Sassone, Giuseppe Fuca', Mario Pedaci, Roberta Lugli, Enrico Bertagnin, Santo Virzi', Manuela Bovina, Giovanni Pasanisi, Simona Mandini, Jonathan Myers, Paolo Tolomeo

**Affiliations:** 1 Division of Provincial Cardiology Department of Translational Medicine University of Ferrara Ferrara Italy; 2 Division of Provincial Cardiology Cardiothoracic Vascular Department Azienda Unità Sanitaria Locale di Ferrara Ferrara Italy; 3 Cardiac Rehabilitation Unit Cardiothoracic Vascular Department Azienda Unità Sanitaria Locale di Ferrara Ferrara Italy; 4 Centre for Exercise Science and Sport Department of Neuroscience and Rehabilitation University of Ferrara Ferrara Italy; 5 Division of Cardiology VA Palo Alto Health Care System Palo Alto, CA United States; 6 Stanford University School of Medicine Stanford, CA United States

**Keywords:** telemedicine, digital literacy, digital health, acute coronary syndrome, older age, caregiver, socioeconomic, educational, mobile phone

## Abstract

**Background:**

Although telemedicine has been proven to have significant potential for improving care for patients with cardiac problems, there remains a substantial risk of introducing disparities linked to the use of digital technology, especially for older or socially vulnerable subgroups.

**Objective:**

We investigated factors influencing adherence to a telemedicine-delivered health education intervention in patients with ischemia, emphasizing demographic and socioeconomic considerations.

**Methods:**

We conducted a descriptive, observational, prospective cohort study in consecutive patients referred to our cardiology center for acute coronary syndrome, from February 2022 to January 2023. Patients were invited to join a web-based health educational meeting (WHEM) after hospital discharge, as part of a secondary prevention program. The WHEM sessions were scheduled monthly and used a teleconference software program for remote synchronous videoconferencing, accessible through a standard computer, tablet, or smartphone based on patient preference or availability.

**Results:**

Out of the 252 patients (median age 70, IQR 61.0-77.3 years; n=189, 75% male), 98 (38.8%) declined the invitation to participate in the WHEM. The reasons for nonacceptance were mainly challenges in handling digital technology (70/98, 71.4%), followed by a lack of confidence in telemedicine as an integrative tool for managing their medical condition (45/98, 45.9%), and a lack of internet-connected devices (43/98, 43.8%). Out of the 154 patients who agreed to participate in the WHEM, 40 (25.9%) were unable to attend. Univariable logistic regression analysis showed that the presence of a caregiver with digital proficiency and a higher education level was associated with an increased likelihood of attendance to the WHEM, while the converse was true for increasing age and female sex. After multivariable adjustment, higher education level (odds ratio [OR] 2.26, 95% CI 1.53-3.32; *P*<.001) and caregiver with digital proficiency (OR 12.83, 95% CI 5.93-27.75; *P*<.001) remained independently associated with the outcome. The model discrimination was good even when corrected for optimism (optimism-corrected C-index=0.812), as was the agreement between observed and predicted probability of participation (optimism-corrected calibration intercept=0.010 and slope=0.948).

**Conclusions:**

This study identifies a notable lack of suitability for a specific cohort of patients with ischemia to participate in our telemedicine intervention, emphasizing the risk of digital marginalization for a significant portion of the population. Addressing low digital literacy rates among patients or their informal caregivers and overcoming cultural bias against remote care were identified as critical issues in our study findings to facilitate the broader adoption of telemedicine as an inclusive tool in health care.

## Introduction

Despite progress in coronary revascularization and pharmacological therapies for acute coronary syndrome (ACS) [1], the residual risk of subsequent major cardiovascular events remains notable, with a 1-year incidence of 18% [2,3]. Implementing secondary prevention strategies in patients with ischemia, involving lifestyle modifications, control of modifiable risk factors, and enhanced adherence to pharmacological therapy, significantly reduces the risk of subsequent cardiovascular events and mortality [4-12].

Since 2015, a standardized follow-up model for recent patients with ACS has been implemented at the Department of Provincial Cardiology of Ferrara. A health education session, initially conducted in person 30 days after discharge as part of a secondary prevention program, was interrupted due to the COVID-19 lockdown imposed by the Italian government in March 2020. In compliance with pandemic measures, in-person meetings were suspended during the mandatory in-home confinement period for most Italian residents. To sustain the provision of our service, we launched a telemedicine development project. This involved health education sessions delivered through videoconferencing appointments, such as web-based remote counseling. Over the last decade, digital health interventions have been proven to be very effective in post-ACS management [13]. However, individuals with lower general or digital literacy may encounter difficulties accessing web-based health care programs, raising concerns about the equitable availability of digital health services for all patients.

Our study aimed to characterize individuals declining or facing barriers in accessing a web-based health education intervention among patients after ACS recently discharged from our cardiology department. We specifically examined demographics and socioeconomic factors influencing adherence to this telemedicine intervention. Identifying potential barriers to digital health care services informs hypotheses to improve accessibility to and use of this emerging branch of health care.

## Methods

### Ethical Considerations

This study was conducted at the Department of Provincial Cardiology of Ferrara, Italy, and was approved by the local Ethics Committee of Area Vasta Emilia Centro (69/2022/Oss/AOUFe; January 20, 2022). Patients’ data were anonymized or deidentified to ensure privacy and confidentiality. The study conformed to the principles of the Declaration of Helsinki. Written informed consent was obtained from all eligible patients before entering the study.

### Patient Selection and Study Design

We conducted an observational, prospective cohort study that included all consecutive, unselected adult (older than 18 years) patients referred to our tertiary cardiology center and discharged with a diagnosis of ACS from February 2022 to January 2023. At the time of hospital discharge, patients who agreed to participate in the study were initially encouraged to complete an exploratory questionnaire designed to assess their proficiency in basic digital technology using a self-assessment scale (from 1 [none] to 10 [very high]), the availability of digital devices for internet connection, and their confidence in telemedicine as a health care resource for their medical condition. When needed, patients were assisted by health care staff dedicated to the study, possibly with the presence of informal caregivers. Second, they were invited to join a free-of-charge web-based health educational meeting (WHEM), as part of a secondary prevention program for cardiovascular diseases.

### Telemedicine Intervention

The WHEM sessions were scheduled monthly using the Lifesize (Lifesize Inc) teleconference software program for remote synchronous videoconferencing while meeting legal requirements to guarantee the security of patients’ data. Patients were asked to participate, preferably 1 month after discharge, using a computer, tablet, or smartphone based on their preference or availability. Patients who agreed to join the WHEM were sent, via email to the address they had provided for communications, the internet link, number code, and instructions to log into the meeting room from anywhere, even anonymously if preferred by the patient. The meeting lasted about 60 minutes and was organized as follows. Initially, an informative presentation was conducted by a properly trained nurse who enforced the basics of healthy lifestyle measures, including smoking cessation, exercise training, and a healthy diet, as well as the importance of therapy adherence and correct medication intake (eg, therapy duration and target doses). We also provided a comprehensive list of commonly asked questions from patients, covering topics such as sexual activity, air travel, and high-altitude activities, along with their respective answers. The presentation was enhanced by visual support in PowerPoint (Microsoft Corporation) to improve communication clarity. Then, a final discussion was held where participating patients had the opportunity to ask questions to health care professionals (eg, cardiologist, kinesiologist, and nutritionist) for a special in-depth focus.

### Data Collection

The following data were collected prospectively, either as continuous or categorical variables—demographic data and geocoded information, educational level, occupational status, number of household members, age of the youngest household member, presence and availability of caregiver with digital literacy, smoking habits, and use of psychotropic medications. Data from patients who declined to participate in the WHEM, along with their reasons for refusal, were documented in the study electronic log and categorized as either related or unrelated to technological literacy in handling the digital aspects of the service. Furthermore, patients who initially agreed to participate but did not follow through were contacted again, and their reasons were collected and grouped into the same categorization as mentioned above for patients who had declined to participate in the WHEM. The education level of the patients was coded and reported as follows—0 for elementary school or no education, 1 for middle school, 2 for high school, and 3 for university. Finally, we mapped and identified patients residing in remote and deprived areas of the province of Ferrara. These territories, spanning 7.078 km^2^ with a population of 55.370 residents, are encompassed within the national strategy for the development of depressed and underserved areas, due to their distinctive conditions identified by the Italian government, and marked by socioeconomic disadvantages resulting from aging processes and demographic decline among the inhabitants [14]. This situation is further compounded by isolation caused by insufficient infrastructure that connects patients to reference centers for the provision of health care services.

Data regarding patients’ demographic and residency details were retrieved from the National Health Information System or the electronic health record, both managed by regional health authorities. Data concerning the remaining variables were acquired through physician or nurse-assisted face-to-face interviews with patients. If patients were unable to provide the required information independently, they received assistance from their caregivers. The technical requirements needed to classify a patient or a caregiver as digitally skilled were derived from self-assessment questions, which aimed to verify possession of a digital device capable of videoconferencing, as well as the ability to effectively use it to access and navigate the internet.

### Statistical Analysis

Our population was categorized according to the participation in the WHEM session. Baseline characteristics are reported as median and IQR (continuous variables) or number and percentages (binary or categorical variables). Differences among groups were tested using the chi-square test for independence and Kruskal-Wallis tests for categorical and continuous variables, respectively. The association between participation in the WHEM session and sex, education level age, and the presence of a caregiver with digital literacy was analyzed using a logistic regression model. The education level of the patients was classified as described in the previous paragraph. Age was modeled as restricted cubic spline with 3 knots placed in the standard position [15]. For each variable included in the logistic regression model, odds ratio (OR), chi-square statistics, and *P* values are reported for both the unadjusted and adjusted model. We further explored the possibility of predicting the final participation in the WHEM session using the multivariable model including variables of interest (sex, age, education, and caregiver with digital literacy). The probability of participating was calculated by applying the logistic function to the linear predictors estimated using the logistic model. The performance measures of this model are reported in terms of discrimination (C-index), overall performance (*R*^2^), and calibration (intercept, slope, and calibration plot). The model was internally validated using a bootstrap-based resampling approach with 2000 repetitions. C-index, *R*^2^, and calibration intercept and slope are reported along with their CIs or optimism corrected, and a smooth calibration plot is also provided [16]. All analyses were performed using R statistical software (version 4.3.2; R Core Team). A *P* value less than .05 was considered statistically significant.

## Results

### Overall Population

After excluding 7 patients due to sensory and cognitive decline that prevented their participation in the investigation, a total of 252 eligible patients completed the questionnaire and were included in the analysis (Multimedia Appendix 1). The median age of the study population was 70 (IQR 61.0-77.3) years, with three-quarters (189/252, 75%) being male. The majority (172/252, 68.2%) of the sample had a low to middle level of education, and approximately two-thirds (168/252, 66.7%) were retired. The overall patients’ characteristics are summarized in Table 1 (overall column).

Upon hospital discharge, 154 (61.1%) out of 252 patients agreed to participate in the WHEM session. Among these 154 patients, only 114 (74%) actually participated in the planned videoconference, while the remaining 40 patients did not follow through. In 21 (52.2%) of the 40 cases, the reason for nonparticipation in the scheduled meeting was difficulty in navigating the videoconference connection procedure. This challenge arose from limited technological literacy or insufficient digital devices for remote videoconferencing. Regarding the 98 (38.9%) out of 252 patients who declined the invitation, 70 (71.4%) of the 98 patients reported general difficulty in handling digital technology as the reason for nonacceptance, with 43 (43.8%) also citing a lack of internet-enabled devices. Additionally, 45 (45.9%) of them expressed a lack of confidence in telemedicine as an integrative tool for managing their medical condition (Multimedia Appendix 2).

Although patients coming from deprived areas were younger (median age 66 vs 71 years; *P*=.04), the mean and distribution of baseline characteristics were balanced across residing areas (Multimedia Appendix 3).

**Table 1:** Characteristics of the study patients.

Variables^a^			Overall (n=252)		Nonparticipants in the WHEM^b^ (n=137)		Participants in the WHEM (n=115)		*P* value	
Age (years), median (IQR)			70.0 (61.0-77.3)		72.0 (65.0-79.0)		68.0 (58.0-75.0)		.001	
Female, n (%)			63 (25)		42 (30.6)		21 (18.2)		.02	
Household members, median (IQR)			2.0 (2.0-3.0)		2.0 (2.0-3.0)		2.0 (2.0-3.0)		.049	
Youngest family member (years), median (IQR)			60.0 (40.8-72.0)		63.0 (46.0-73.0)		52.0 (32.0-70.0)		.01	
Residents in deprived areas, n (%)			45 (17.8)		27 (19.7)		18 (15.6)		.40	
**Educational level, n (%)**										<.001
	Elementary school or no education	79 (31.3)		63 (45.9)		16 (13.9)				
	Middle school	93 (36.9)		46 (33.5)		47 (40.8)				
	High school	63 (25)		25 (18.2)		38 (33)				
	University	17 (6.7)		3 (2.1)		14 (12.1)				
Actively employed worker, n (%)			84 (33.3)		36 (26.2)		48 (41.7)		.01	
Caregiver with digital literacy, n (%)			164 (65.1)		59 (43.1)		105 (91.3)		<.001	
User of antidepressant drugs, n (%)			31 (12.3)		16 (11.6)		15 (13)		.74	
Active smoker, n (%)			64 (25.3)		29 (21.1)		35 (30.4)		.09	

^a^All continuous variables are presented as median and IQR and tested using the Wilcoxon test. Categorical variables are reported as numbers and percentages (%) and tested using the Fisher test.

^b^WHEM: web-based health educational meeting.

### Baseline Characteristics According to Participation in the WHEM

Out of 252 patients, 115 (45.6%) attended the WHEM session (Multimedia Appendix 4), including 1 who had initially declined. According to Table 1 (participants’ column), overall, patients who participated tend to be younger and have a higher level of education. They were also more likely to be male, active workers, and supported by caregivers with digital proficiency. Conversely, no difference was found for residing areas when compared with nonparticipants.

### Predictors of Participation in the WHEM

The results of the logistic regression models (univariable and multivariable) are shown in Table 2. From the univariable logistic regression analysis, the presence of a caregiver with digital proficiency and a higher education level was associated with an increased likelihood of attending the provided digital health service, while the opposite was true for increasing age and female sex. After multivariable adjustment, higher educational levels and caregivers with digital skills remained independently associated with the outcome.

The probability of participation varied by education level, analyzed both unadjusted (univariable) and adjusted for age, sex, and the presence of a caregiver with digital literacy (multivariable). In the univariable analysis, among individuals with elementary school education or none, 79.7% (63/79) were participants and 20.3% (16/79) were nonparticipants; for those with middle school education, 49.5% (46/93) were participants and 50.5% (47/93) were nonparticipants; in the high school education group, 39.7% (25/63) were participants and 60.3% (38/63) were nonparticipants; and among those with university education, 17.6% (3/17) were participants while 82.4% (14/17) were nonparticipants. In the multivariable analysis, for individuals with elementary school education or none, the adjusted number of participants was estimated at 96.2% (76/79), with 3.8% (3/79) being nonparticipants; among those with middle school education, the estimated number of participants was 86% (80/93) and nonparticipants was 14% (13/93); in the high school education group, the estimated number of participants was 81% (51/63) and nonparticipants was 19% (12/63); and for individuals with university education, the estimated number of participants was 58.8% (10/17) and nonparticipants was 41.2% (7/17). These results indicate a higher probability of participation among individuals with lower levels of education when additional factors such as age, sex, and the presence of a digitally literate caregiver are considered in the analysis. This contrasts with the raw, unadjusted proportions observed in the univariable analysis.

**Figure 1:**
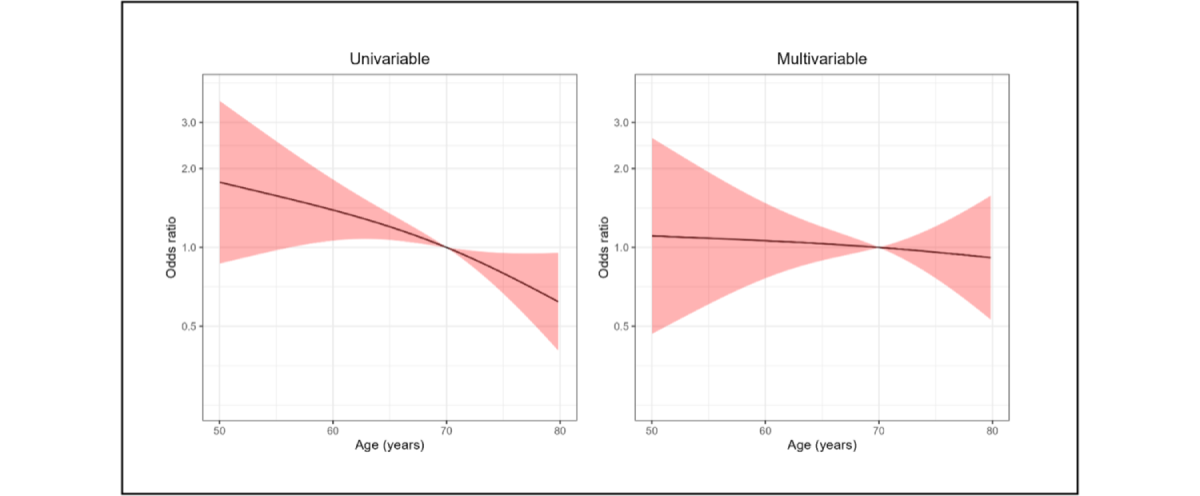
The odds ratio (OR) of the binary outcome (participation, yes or no) according to age. The outcome is modeled using logistic regression and age is included as restricted cubic spline with 3 knots. The effect (OR) is reported unadjusted (univariable panel) and adjusted for sex, education level, and the presence of a caregiver with digital literacy (multivariable panel). The black line and red shaded area represent point estimates (OR) and 95% CI, respectively.

As graphically presented in Figure 1, the multivariable adjustment mitigated the unadjusted effect of age on participation probabilities, as highlighted by a mainly flat shape of the OR across the continuous variable “age.”

The performance measures of the multivariable model (as a model to predict the participation) are reported in Table 3. The model discrimination was good even when corrected for optimism (optimism-corrected C-index=0.812), as was the agreement between observed and predicted probability of participation (optimism-corrected calibration intercept=0.010 and slope=0.948).

**Figure 2:**
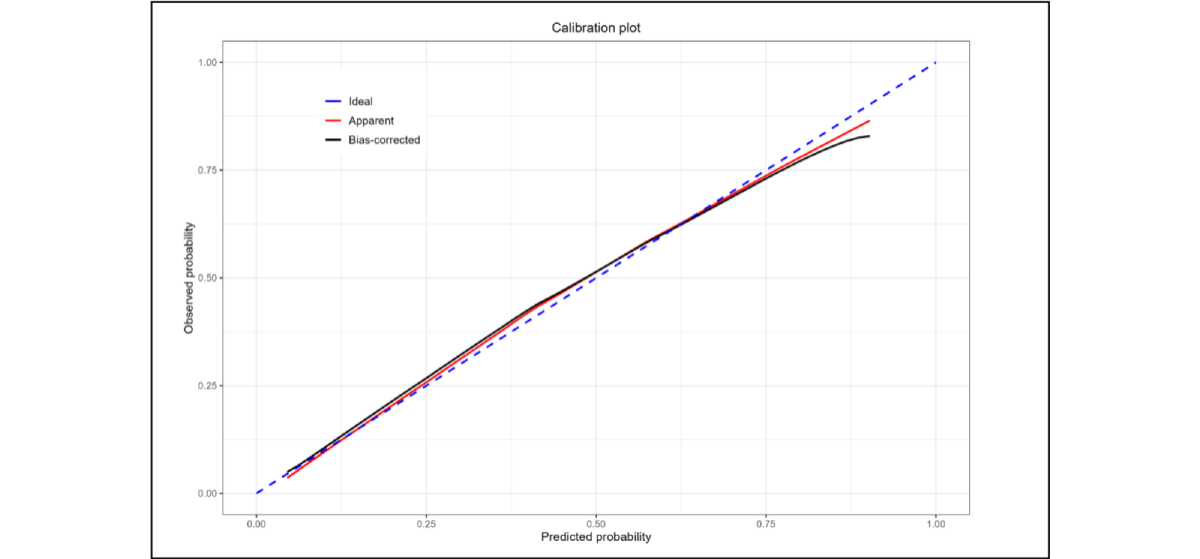
The agreement between actual (observed, y-axis) and predicted (x-axis) probability of participation. The probability is predicted using a logistic regression model including age, sex, education level, and the presence of a caregiver with digital literacy. The dashed blue line represents a perfect calibration. Red and black lines represent apparent and optimism-corrected calibration (using a bootstrap approach with 2000 replications), respectively.

Moreover, the agreement across the entire range of predicted probability is shown in Figure 2 as a smooth calibration plot. The equation of the fitted model is reported in the supplementary materials (Multimedia Appendix 5).

**Table 2:** Logistic regression model^a^.

Variables	Univariable			Multivariable		
	OR^b^ (95% CI)	Chi-square (*df*)	*P* value	OR (95% CI)	Chi-square (*df*)	*P* value
Sex (female)	0.51 (0.28-0.92)	5.2 (1)	.02	0.92 (0.44-1.92)	0.1 (1)	.83
Educational level	2.43 (1.77-3.34)	35.3 (1)	<.001	2.26 (1.53-3.32)	18.7 (1)	<.001
Digital-skilled caregiver	13.88 (6.68-28.85)	70.8 (1)	<.001	12.83 (5.93-27.75)	57.4 (1)	<.001
Age (years)	0.55 (0.38-0.79)	10.6 (1)	.001	0.90 (0.55-1.45)	0.2 (1)	.66

^a^The binary outcome of each the model is the participation (yes or no). Odds ratio for age is reported as IQR (from 61 to 77.25 years). For each variable, the chi-square statistic and corresponding *P* values from a likelihood ratio test are reported.

^b^OR: odds ratio.

**Table 3:** Performance measures^a^ of the multivariable model.

	C-index (95% CI)	*R* ^2^	Calibration intercept	Calibration slope
Apparent	0.820 (0.770-0.870)	0.420	0.000	1.000
Optimism corrected	0.812 (0.770-0.874)	0.398	0.010	0.948

^a^Apparent C-index is reported along with 95% CI. Overall performance is reported as *R*^2^. Calibration is reported as calibration intercept and slope. Optimism-corrected measures are estimated using a bootstrap approach with 2000 replications.

## Discussion

### Principal Findings

Our study investigated demographic and socioeconomic factors that affected adherence to a WHEM in patients recently diagnosed with ACS. This diagnosis included a spectrum of medical emergencies caused by coronary hypoperfusion resulting in myocardial tissue injury, ranging from transient myocardial ischemia to cell death in cases of prolonged ischemia [17]. This patient population has specific health care needs and challenges in postdischarge care. The residual risk of cardiovascular events underscores the importance of secondary prevention strategies, including lifestyle modifications and adherence to pharmacological therapy. Generally, post-ACS follow-up care is commonly provided in outpatient settings by cardiologists and specialized nurses. The main finding of our study can be summarized as follows: first, slightly more than half of the eligible patients did not participate in our telemedicine intervention; second, a notable proportion of patients declined the invitation to participate, primarily due to a lack of adequate literacy or confidence in digital health solutions; third, more than a quarter of those who initially consented to participate in the WHEM failed to access the scheduled session, with challenges in handling digital technology being the leading cause; and finally, patient’s educational level and the presence of a caregiver with digital proficiency were independently associated with the ability to access the WHEM.

### Comparison With Prior Work

In 2018, the World Health Organization promoted telemedicine as a method for delivering secure and cost-effective care to underserved populations [18]. However, technology itself may act as a deterrent for patients lacking either sufficient digital literacy or technologically suitable personal mobile devices for remote connection. In a survey of 1604 mobile phone users across the United States, nearly 46% of individuals who had installed a health-related app discontinued its use. The majority of them cited a significant burden of data entry or complexity in navigating web-based services as the primary reasons for their decision [19]. In our study, despite the relatively simple connection process for accessing the health-related web-based service, 36.1% (91/252) of patients (21/154, 13.6% of willing participants and 70/98, 71.4% of unwilling participants) could not proceed due to technological-related challenges. Similarly, in a randomized and controlled trial carried out in the Netherlands and designed to assess the feasibility of a smart technology-mediated intervention for blood pressure control among patients after ACS, the 33% nonparticipation rate in the trial was linked to a fear of not being able to cope with technology [20]. In a recent Italian survey assessing digital literacy among unselected patients attending a tertiary cardiology outpatient clinic, 42% of patients reported never accessing the internet [21]. Although the authors did not investigate the reasons for this widespread lack of confidence in digital solutions, demographic, socioeconomic conditions, or educational background have been speculated as likely factors responsible for it. While the number of regular internet users has been increasing worldwide, specific demographics, such as older adults and individuals with lower household income, have been found to be less likely to own devices for accessing the internet [22-24]. In our study, 17.1% (43/252) of patients reported not having appropriate devices for videoconferencing through an internet connection. Similar findings, in a comparable clinical setting, emerged from an Egyptian randomized controlled trial investigating the impact of telemedicine on short-term follow-up for patients admitted with acute myocardial infarction [25]. In that trial, 14% of patients could not participate due to the absence of smartphones suitable for videoconferencing and an additional 10% were excluded due to a lack of internet connectivity. Moreover, older age has consistently arisen as a factor for diminished internet accessibility, decreased use of digital health technology, and greater challenges in engaging with digital health care services across numerous studies [26,27]. In our study, the significantly lower median age of the youngest family member in households of participating patients (52 vs 63 years; *P*=.01) was likely associated with the substantially higher availability of a caregiver with digital competence, more than doubling the participation rate compared to nonparticipating patients. In turn, the presence of a caregiver with digital proficiency emerged as the strongest independent predictor of participation in our telemedicine intervention. A report from the Italian National Institute of Statistics conducted nationwide in 2023 shows that in households consisting solely of older individuals (65 years of age or older), slightly more than half (53.4%) have access to the internet, compared to 93.6% of households with members who are not solely older. Among such older households, 67% report a lack of digital skills as the reason preventing them from connecting to the internet. Therefore, given the global aging population along with the growing importance of digital technology in supporting conventional health care, informal caregivers with digital skills become key contributors to the sustainability of social and health care systems. According to Hoffmann and Rodrigues [28], it is estimated that approximately 80% of long-term care in Europe is delivered by informal caregivers. The currently available estimates for the prevalence of informal caregivers in Europe range from 10% to 25% of the total population [29]. As a consequence, it would be strategic to provide telemedicine knowledge for informal caregivers who lack it. Furthermore, given the advanced age of our patient cohort, with one-quarter (63/252, 25%) being older than 77 years old, achieving and improving digital literacy among individuals who provide informal care is likely to be more feasible and effective than focusing solely on the dependent older adults they care for.

A population-based Australian survey of adults indicated that each decade of higher age was linked to a 20% reduction in the odds of engagement with the national web-based personal health care record [30]. A large US study, conducted during the peak of the COVID-19 pandemic in 2020, revealed inequities in telemedicine use for health care in cardiology, with older age being associated with a lower use of video for remote visits compared to individuals younger than 55 years of age [31]. In our study, univariable logistic regression analysis revealed that the age of patients within the IQR of 61-77 years was associated with a decreased likelihood of participation in the WHEM, with the odds declining by almost 50% from the first to the third quartile (Table 2). Although the multivariable analysis suggests increased uncertainty in estimating this association, the small sample size included in the logistic regression model likely influenced these results.

Similar to older age, the literature suggests that lower levels of education are linked to lower proficiency in using web-based technologies and reduced access or less use of the internet for health care–related activities [32-36]. Data from a large, nationally representative survey of the noninstitutionalized adult population in the United States showed that the level of education strongly predicted internet usage for engaging in health care–related activities and services, with patients having lower levels of education less likely to use technology compared to those with higher education attainment [37]. A register-based study conducted on the entire population of Stockholm revealed that the likelihood of engaging in telemedicine consultations decreased with lower levels of educational attainment [38]. Consistent with these findings, our study revealed a significantly lower average education level score among nonparticipating patients compared to participating patients (0.77, SD 0.82 vs 1.43, SD 0.88; *P*<.001), with a notably higher percentage of patients with only elementary school education or no formal education among the nonparticipating patients (63/137, 45.9% vs 16/115, 13.9%).

The gender disparity in digital literacy and access to digital technologies has been recognized since the 2000s, with women being less represented. However, despite this disparity decreasing [39,40], findings on the digital gender gap in telemedicine remain conflicting across studies. In the abovementioned US study, female sex was associated with less use of videoconferencing for telemedicine interventions in specialty care, including cardiology [22]. Conversely, a survey of the Israeli adult population conducted in 2008 found no relationship between gender and the ability to use telemedicine for health care-related activities [41]. These findings aligned with the study conducted by Mizrachi et al [42], which revealed that the digital divide index among gender categories in an Israeli sample was smaller than that compared to other factors like education, household income, and age. In our cohort, the rate of female participants was almost twice as high in nonparticipating patients compared to participating patients. However, although univariably significant when considered as a crude variable, female sex was not independently associated with a lower likelihood of participating in WHEM.

The catchment area covered by our study is characterized by socially disadvantaged groups of patients living in remote and underserved areas. While these areas might be ideal for telemedicine interventions, residing in underserved areas was associated with reduced internet access due to socioeconomic conditions and demographic characteristics [43-46]. In our study, although the participation rate in WHEM was not different between residents in deprived and nondeprived areas, the limited sample size does not allow us to draw definitive conclusions.

### Interventions for Telemedicine Engagement

We investigated the potential to predict future participation in WHEM using a multivariable model that included sex, age, education, and the presence of a caregiver with digital literacy (Multimedia Appendix 5). Predicting the individual probability of participation could enable clinicians to customize engagement strategies and education programs on telemedicine. To validate our model, we used a resampling technique (bootstrap). When the predicted probability is low (based on a clinically meaningful cutoff), patients could receive an in-person–streamlined education intervention during hospitalization or shortly after discharge. This intervention aims to address cultural biases against remote care and provide basic training for using common devices to connect to the internet and access specific software. It is worth noting that the digital literacy of the caregiver, which strongly predicted participation, can be acted upon and improved with targeted training if needed. Health care professionals with digital skills will lead training sessions, possibly supported by information and communication technologies experts. Personalized technical support could also be provided to patients at their homes by the family-community nurse, a recently introduced key role in the Italian National Health Service focused on promoting health and managing chronic conditions within the community. These nurses also assist by providing mobile digital-enabled devices. Furthermore, skill training could be scheduled and offered to patients and their informal caregivers at net-point facilities in nonhospital health care centers, enhancing the community and service-market fit of our telemedicine intervention.

### Limitations

Some limitations need to be addressed in this study. First, an inherent limitation of our study is its observational nature, which may introduce potential biases such as selection bias and confounding variables that cannot be fully controlled for in the absence of randomization. Second, a major limitation is the relatively small sample size of the study population, which might have affected the statistical results, especially during subgroup analysis. Third, data concerning reasons for the refusal of the telemedicine intervention were limited to either related or unrelated to digital proficiency. Therefore, personal or motivational factors influencing their refusal have been not assessed. Fourth, to assess proficiency in the domain of digital technology within our study population, we used a nonvalidated questionnaire, partially relying on a self-assessment scale. Consequently, the absence of standardized criteria may not have accurately captured the true digital skills of the population, potentially introducing bias in the results and, as a result, making it challenging to compare our findings with those of similar research. Moreover, results derived from self-appraisals heighten the risk of unreliable or inconsistent results due to the inherently self-referential nature of responses. These in turn may be influenced by educational levels and cognitive status, potentially compromising comparability across patients. Finally, our findings were derived from studying a population within a specific clinical context, such as recent ACS, which might have transiently influenced the psychological state of patients, potentially impacting their willingness to engage in a nonstandardized health care approach such as telemedicine interventions. These results may not be replicable when applied to patients in different clinical contexts, such as those with chronic conditions.

### Conclusions

While telemedicine has been proven to have significant potential for improving patient care, the persistent risk of introducing disparities linked to the use of digital technology remains substantial, especially when examining subgroups within either older or socially vulnerable populations. In this study, the telemedicine intervention we provided within a secondary cardiovascular prevention program proved impractical for a substantial group of patients with ischemia. This highlights a distinct lack of suitability for this specific cohort and underscores the risk of digital marginalization for a significant proportion of the population. Low digital literacy rates and a cultural bias against remote care have been the main barriers responsible for nonparticipation. We are aware that our analysis remains descriptive in nature and should be contextualized within a patient cohort from the province of Ferrara, characterized by both one of the highest aging samples in Italy and a significant proportion of households comprised exclusively of older individuals. Therefore, the upcoming challenge will involve developing solutions to bridge the existing gap that hinders the integration of digital solutions as a routine aspect of health care delivery in cardiology. Our results should be considered hypothesis generating, paving the way to explore new organizational models oriented toward a patient-centered, community-based health care approach. Building on this perspective, we provided the full equation of our simple 4-variable model for predicting individual participation probabilities. This step is critical to enable external research groups to apply the model to their own populations, and we encourage them to validate and potentially refine our findings.

## Appendix

Multimedia Appendix 1 Flowchart diagram showing the selection and exclusion process of the initial population, and the final study group of patients.

Multimedia Appendix 2 Reasons for declining the invitation to participate in the web-based health educational meeting.

Multimedia Appendix 3 Patient characteristics according to residing areas.

Multimedia Appendix 4 Contingency table: 2x2 relationships.

Multimedia Appendix 5 Multivariable model predicting individual participation in the web-based health educational meeting.

## Abbreviations

ACS: acute coronary syndrome
OR: odds ratio
WHEM: web-based health educational meeting
