# Supplementary figures and images for: Analysis of Demographic and Socioeconomic Factors Influencing Adherence to a Web-Based Intervention Among Patients After Acute Coronary Syndrome: Prospective Observational Cohort Study
Source: JMIR Cardio. 2024 Aug 2;8:e57058. doi: 10.2196/57058 (PMC11329845; doi:10.2196/57058)

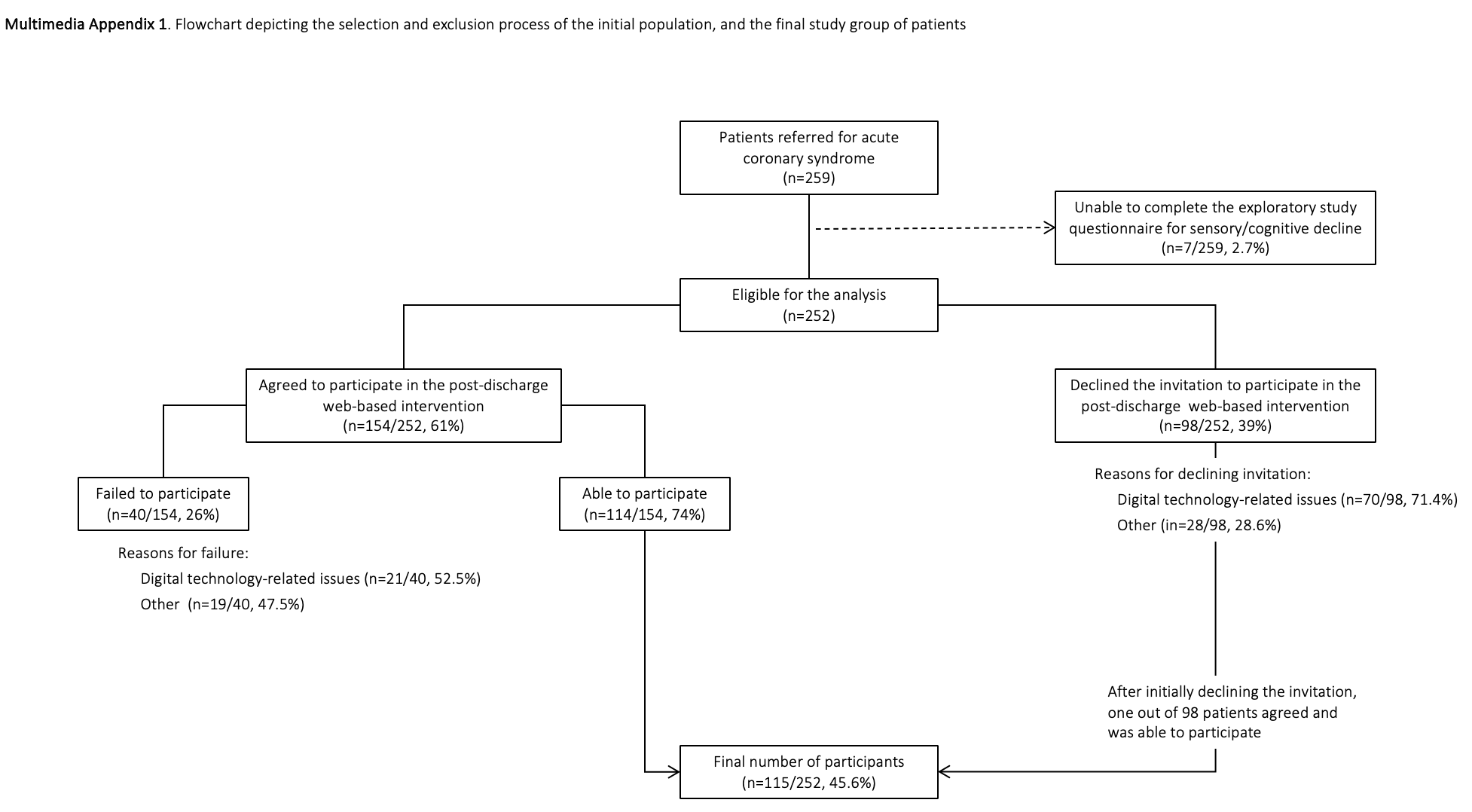

Supplement: Multimedia Appendix 1 [file cardio_v8i1e57058_app1.docx]

The probability of participation can be estimated as follows:


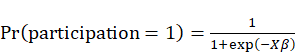


where Xb is defined as


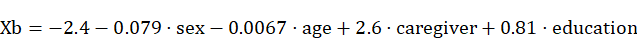

Supplement: Multimedia Appendix 5 [file cardio_v8i1e57058_app5.doc]
