# Supplementary material for: Analysis of Demographic and Socioeconomic Factors Influencing Adherence to a Web-Based Intervention Among Patients After Acute Coronary Syndrome: Prospective Observational Cohort Study
Source: JMIR Cardio. 2024 Aug 2;8:e57058. doi: 10.2196/57058 (PMC11329845; doi:10.2196/57058)
Supplement: Multimedia Appendix 2 [file cardio_v8i1e57058_app2.doc]

###

| Reasons for declining the invitation | Patients declining the invitation (n=98) |
| --- | --- |
|  |  |
| General difficulty in handling digital technology | 70 (71.4%) |
|  |  |
| Lack of device for Internet connection | 43 (43.8%) |
|  |  |
| Lack of digital skills | 53 (54%) |
|  |  |
| Lack of confidence in telemedicine | 45 (45.9%) |
