# Supplementary material for: Analysis of Demographic and Socioeconomic Factors Influencing Adherence to a Web-Based Intervention Among Patients After Acute Coronary Syndrome: Prospective Observational Cohort Study
Source: JMIR Cardio. 2024 Aug 2;8:e57058. doi: 10.2196/57058 (PMC11329845; doi:10.2196/57058)
Supplement: Multimedia Appendix 3 [file cardio_v8i1e57058_app3.doc]

| Variable | Non-participants in web-based  health educational meeting  (n=137/252, 54.3%) | Participants in web-based  health educational meeting  (n=115/252, 45.7%) |
| --- | --- | --- |
|  |  |  |
| Willing to participate |  |  |
|  |  |  |
| No (n=98/252, 39%) | 97 (99%) | 1 (1%) |
|  |  |  |
| Yes (n=154/252, 61%) | 40 (26%) | 114 (74%) |

### 
